# ATP reduces mitochondrial MECR protein in liver of diet-induced obese mice in mechanism of insulin resistance

**DOI:** 10.1042/BSR20200665

**Published:** 2020-06-04

**Authors:** Shengnan Qian, Li Ma, Shiqiao Peng, Yanhong Xu, Kaiyue Wu, Shuang Shen, Xiaoying Zhang, Yongning Sun, Jianping Ye

**Affiliations:** 1 College of Food Science and Technology, College of Fisheries and Life Science, Shanghai Ocean University, Shanghai 201306, China; 2 Central Laboratory, Shanghai Sixth People’s Hospital East Campus, Shanghai University of Health and Medical Sciences, Shanghai 201306, China; 3 Department of Traditional Chinese Medicine, Shanghai Jiao Tong University Affiliated Sixth People’s Hospital, Shanghai 200233, China; 4 Department of Cardiology, Shanghai Municipal Hospital of Traditional Chinese Medicine, Shanghai University of Traditional Chinese Medicine, Shanghai 200071, China; 5 Shanghai Diabetes Institute, Shanghai Jiao Tong University Affiliated Sixth People’s Hospital, Shanghai 201306, China

**Keywords:** ATP, Berberine, hepatic physiology, insulin resistance, MECR, mitochondrial dysfunction

## Abstract

Mitochondrial 2-enoyl-acyl-carrier protein reductase (MECR) is an enzyme in the mitochondrial fatty acid synthase (mtFAS) pathway. MECR activity remains unknown in the mechanism of insulin resistance in the pathogenesis of type 2 diabetes. In the present study, MECR activity was investigated in diet-induced obese (DIO) mice. *Mecr* mRNA was induced by insulin in cell culture, and was elevated in the liver of DIO mice in the presence hyperinsulinemia. However, MECR protein was decreased in the liver of DIO mice, and the reduction was blocked by treatment of the DIO mice with berberine (BBR). The mechanism of MECR protein regulation was investigated with a focus on ATP. The protein was decreased in the cell lysate and DIO liver by an increase in ATP levels. The ATP protein reduction was blocked in the liver of BBR-treated mice by suppression of ATP elevation. The MECR protein reduction was associated with insulin resistance and the protein restoration was associated with improvement of insulin sensitivity by BBR in the DIO mice. The data suggest that MECR protein is regulated in hepatocytes by ATP in association with insulin resistance. The study provides evidence for a relationship between MECR protein and insulin resistance.

## Introduction

Mitochondria provide intermediate metabolites (such as acetyl-CoA) to the glucose production pathway (gluconeogenesis) in hepatocytes in the maintenance of blood glucose by liver in the fasting conditions. The gluconeogenesis is inhibited by insulin in the postprandial condition to attenuate the meal-induced hyperglycemia. The hepatocyte response to insulin is impaired in the insulin-resistant conditions leading to hyperglycemia, a pathological character of type 2 diabetes. Insulin resistance is a result of energy surplus [1], and ATP is a signal for the energy surplus [2]. Inhibition of ATP production by mitochondria is a mechanism for the diabetic medicine metformin in the improvement of insulin sensitivity [3]. ATP is believed to induce insulin resistance through a feedback regulation of mitochondrial function. ATP holds a promise for unlocking the secret of insulin resistance [4]. However, the molecular target of ATP remains to be identified in mitochondria.

Cross-talk of mitochondrial products is a approach for understanding the feedback regulation of mitochondrial function. In addition to the well-known products such as ATP, α-lipoic acid (LA), a fatty acid derivative of octanoic acid, is a mitochondrial lipid product [5]. LA promotes ATP production from glucose through modification of pyruvate dehydrogenase (PDH) by lipoylation reaction in mitochondria [6] or activation of AMPK [7]. Pyruvate dehydrogenase gains function after lipoylation to promote conversion of pyruvate into acetyl-CoA. There is no information about ATP impact in the LA synthesis. Endogenous LA is produced inside mitochondria by mitochondrial fatty acid synthase (mtFAS or FAS II) in yeast, but the pathway remains to be investigated in mammalian cells [5,6,8]. In early studies, we investigated two enzymes (MCAT and OXSM) in the mtFAS pathway, and found the roles of glucose [9,10], lactic acid [11] and inflammation in the regulation of their activities [10]. Mitochondrial 2-enoyl-acyl-carrier protein reductase (MECR) is an enzyme for production of octanoic acid in the mtFAS pathway [12]. MECR is required for mitochondrial function. MECR dysfunction from gene mutation leads to mitochondrial deficiency in patients [13] and MECR gene deletion generates embryonic lethality in mice [14]. Super induction of MECR activity by gene overexpression leads to an increase in mitochondrial size, but a functional reduction in ATP production [15]. MECR is important for the mitochondrial function in mammalians, but mechanism of MECR regulation remains unknown in the physiological conditions. In the present study, we investigated mRNA and protein expression of MECR in diet-induced obese (DIO) mice in the study of insulin resistance.

In the current study, MECR expression was examined *in vitro* and *in vivo*. mRNA was induced by insulin and protein was reduced in the DIO mice. The protein was restored in the liver by berberine (BBR) treatment of DIO mice, which was associated with attenuation of ATP elevation. MECR represents a target molecule of insulin and ATP in the mechanism of mitochondrial regulation.

## Materials and methods

### Cell culture and treatment

The mouse fibroblast cell line 3T3-L1 (CL-173) and Hepa-1c1c7 cell line (CRL-2026) were purchased from the American Type Culture Collection (Manassas, VA). The cells were cultured in DMEM (SH30243.01, HyClone, 4.5g/l glucose, U.S.A.) supplemented with 10% fetal bovine serum (10270-106, GIBCO, U.S.A.) and 1% antibiotics (15240062, GIBCO, U.S.A.) at 37°C in a 5% CO_2_ incubator. The cells were treated with glucose (35 mM, G8150, Solarbio), TNF-α (10 ng/ml, 315-01A, Peprotech, U.S.A.), insulin (200 nM), forskolin (20 μM) and hypoxia (200 μg/ml sodium sulfite, Na_2_SO_3_, Sigma, St. Louis, MO, U.S.A.) [16]. The stock solutions of the agents were prepared at 1000-times of the final concentrations.

### Animal models

The animal studies were performed according to the animal protocols approved by the Institutional Animal Care and Use Committees (IACUC) at the Shanghai Sixth People’s Hospital, Shanghai Jiao Tong University. C57BL/6 mice (male, 6-week-old) were obtained from the Shanghai Xipuer Bikai Laboratory Animal Co. Ltd. (Shanghai, China). The mice were kept under controlled conditions for temperature (22 ± 2°C), humidity (60 ± 5%) and a 12-h dark/light cycle in the animal facility of Shanghai Jiao Tong University (SPF grade). The lean mice were fed on Chow diet (13.5% calorie in fat; Shanghai Slac Laboratory Animal Co. Ltd) *ad libitum* and DIO mice were fed on a high-fat diet (HFD, 60% calorie in fat; # D12492, Research Diets, U.S.A.) for 16 weeks. The DIO mice were divided randomly into three groups at *n*=12. The DIO control mice were not treated with a drug, and the other two groups were treated for 8 additional weeks with BBR (100 mg/kg/day) [17]. BBR was administrated through dietary supplementation. The liver tissues were collected from the mice after euthanasia with cervical dislocation under anesthesia following an intraperitoneal injection of sodium pentobarbital (35 mg/kg) at the end of treatment.

### ATP impact in MECR protein

ATP concentration was tested by modification of a protocol reported in an early study [18]. ATP in the liver of DIO mice was determined in the fresh tissue using the ATP test kit (A22066, Thermo Fisher Scientific, U.S.A.) according to the manufacturer’s protocol. In the *in vitro* study, the cell lysate was prepared from 1c1c7 cells with the whole cell lysis buffer, and stored at −80°C for overnight to deplete the endogenous ATP. Exogenous ATP (SLBW4494, Sigma, U.S.A.) was added into the cell lysate (3 μg/μl, 10 μl) to elevate ATP levels for different concentrations as indicated in the figure legend. After incubation in 37°C water bath for 15 min, the MECR protein was determined by Western blotting, and ATP was determined with the test kit in the lysate.

### Western blotting

Western blotting was conducted according to a protocol described elsewhere [18]. The antibodies to lipoylation (ab58724) and MECR (ab156268) were purchased from the Abcam Trading Company (Shanghai, China). The protein signal was quantified using the ImageJ program as described in an early study [11].

### qRT-PCR

mRNA was quantified with qRT-PCR according to a protocol reported elsewhere [19]. Total RNA was extracted from the cells or tissues using the RNA Extraction Reagent (R401-01, Vazyme Biotech, China). RNA was quantified with SpectraMax i3× (Molecular Devices) and mRNA (100 ng/μl) was transcribed into cDNA using the HiScript® II Q RT SuperMix for qPCR (+g DNA wiper) (R223, Vazyme Biotech, China). mRNA was quantified with ChamQ™ Universal SYBR qPCR Maser Mix (Q711,Vazyme Biotech) on LightCycle 480 II (Roche). The result was normalized with the *Gapdh* signal. The primer sequences are listed in Table 1. The relative expression level of *Mecr* mRNA was calculated by 2^−ΔΔ*C*_t_^ method.

**Table 1:** Primers used in qRT-PCR for quantification of *Mecr* mRNA

Primer	Sequence
*Mecr* Forward	5′-CTGCATTGAAGCCAGGAGAT-3′
*Mecr* Reverse	5′-GGATTCCAATCAGTGCTTCCT-3′
*Gapdh* Forward	5′-GACGGCCGCATCTTCTTGT-3′
*Gapdh* Reverse	5′-CACACCGACCTTCACCATTTT-3′

The primers were synthesized according to the sequence in the table.

### Fasting insulin and glucose

The vein blood was collected from the mice after overnight fasting (14 h). Insulin and glucose were tested with the Rat Insulin Assay (RIA) kit (Linco Research, St. Charles, Missouri, U.S.A.) and One Touch glucometer (ACCU-CHEK® performa, Roche) using the protocols described elsewhere [17]. The insulin sensitivity index HOMA-IR [= fasting insulin (mU/l) × fasting glucose (mM)/22.5] was calculated according to the fasting insulin and glucose concentration [20].

### Statistical analysis

The results were statistically analyzed with the Student’s *t* test at statistical significance of *P*<0.05. The data are expressed as mean ± standard deviation (SD). All cellular experiments were repeated at least three times with consistent results.

## Results

### mRNA of MECR is induced by insulin and inhibited by forskolin

**Figure 1:**
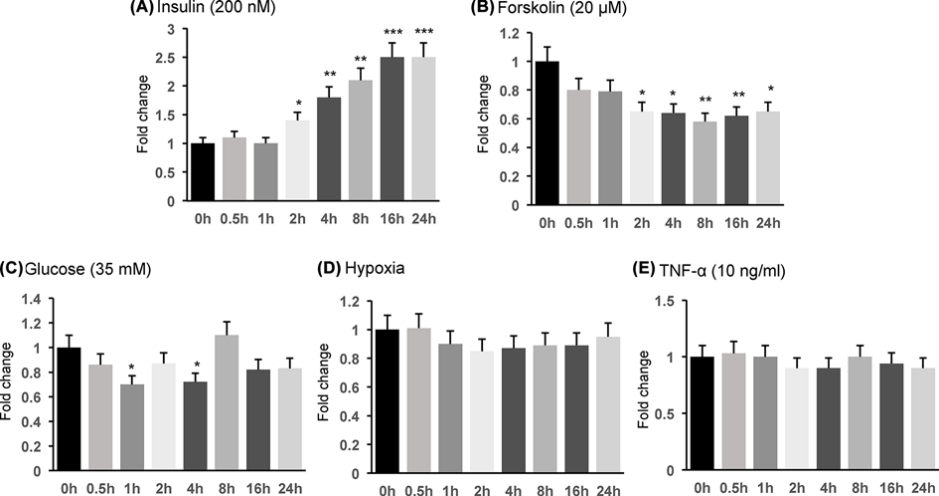
*Mecr* mRNA induction by insulin and inhibition by forskolin (**A**) Induction of *Mecr* mRNA by insulin. Undifferentiated 3L3-L1 cells were used in the study with insulin (200 nM) treatment for 24 h. The expression was determined at multiple time points as indicated by qRT-PCR. Following studies were conducted in the same condition for other factors. (**B**) Inhibition of *Mecr* mRNA by forskolin (20 μM). (**C**) Regulation of *Mecr* mRNA by glucose (35 mM). (**D**) Regulation of *Mecr* mRNA by hypoxia. The hypoxia was induced by addition of sodium sulfite (Na_2_SO_3_, 200 μg/ml) into the cell culture medium. (**E**) Regulation of *Mecr* mRNA by TNF-α (10 ng/ml). The data in bar figure represents mean ± SD (*n*=3). *, *P*<0.05; **, *P*<0.01; ***, *P*<0.001 by Student’s *t* test.

MECR is a nucleus-encoded mitochondrial protein and required for maintenance of the mitochondrial function. However, there was no information about regulation of *Mecr* expression in mammalian cells in the literature. To address this issue, we examined mRNA of *Mecr* in 3T3-L1 cells in response to several stimulations related to insulin resistance. The expression was induced by insulin in a time-dependent manner (Figure 1A), which was observed at 2 h with a peak at 16–24 h. The expression was reduced by activation of the cAMP/PKA signaling pathway with forskolin in a time-dependent manner (Figure 1B). The inhibition appeared at 1 h and reached the peak at 8 h in the presence of forskolin. In response to a high level of glucose (35 mM), the expression exhibited an oscillation, with a reduction at 0.5, 4 and 16 h (Figure 1C). In response to hypoxia or inflammatory cytokine TNF-α, no significant reduction was observed (Figure 1D,E). These data suggest that *Mecr* mRNA is regulated by insulin, forskolin and glucose with different patterns.

### MECR protein abundance in multiple tissues

**Figure 2:**
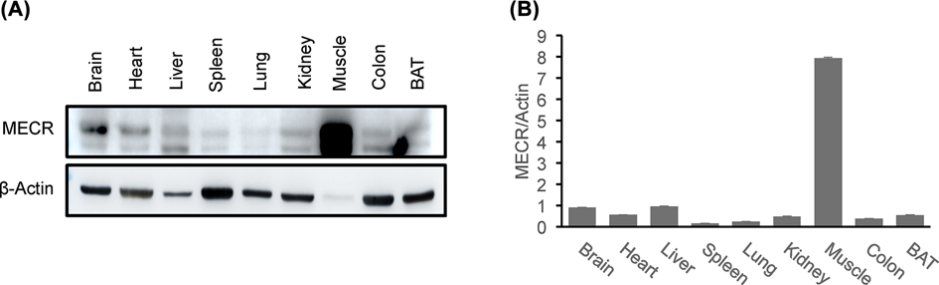
MECR protein expression in mouse tissues (**A**) MECR protein in Western blot. The protein was determined in multiple tissues of normal mice by Western blotting. The experiment was conducted in three mice. A representative image is shown. (**B**) Quantification of the protein in Western blots. The protein signal was quantified in three blots and the mean value was expressed in the bar figure. The data in bar represent mean ± SD (*n*=3).

MECR is highly expressed in mitochondria in yeast, but there is no report about its protein distribution in the mammalian tissues. To address the issue, abundance of MECR protein was examined in nine mitochondria-enriched tissues (brain, heart, liver, spleen, lung, kidney, skeletal muscle, colon and brown fat) in mice by Western blotting. Based on the expression level with β-actin normalization, these tissues were organized into four groups. The protein abundance was most abundant in the skeletal muscle, which was approximately seven-times higher than those in the second group of tissues including brain, heart and liver (Figure 2A,B). The abundance of third group including kidney, colon and brown fat was approximately half of that in the second group. The lowest abundance was found in the fourth group of spleen and lung, in which the expression was approximately half of that in the third group. The results suggest that MECR protein is expressed in all tissues with different abundance, suggesting that the protein may be regulated in a tissue-specific manner.

### MECR protein is decreased in liver by obesity

**Figure 3:**
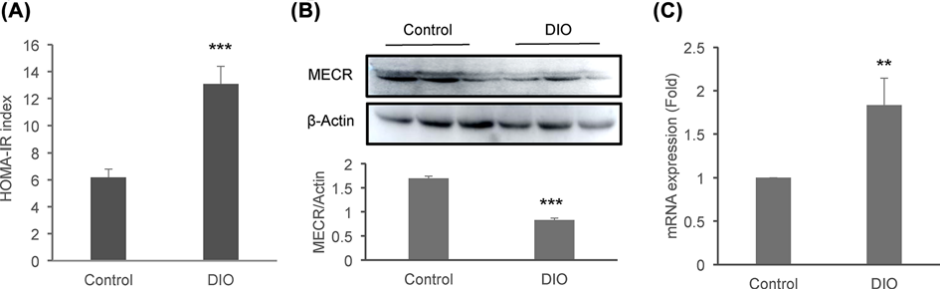
Reduction in MECR protein in liver of DIO mice (**A**) Insulin sensitivity. The insulin resistance index of HOMA-IR was calculated with fasting insulin and fasting glucose using the formula listed in the method. (**B**) MECR protein in Western blot. Each lane represents one mouse. The signal was quantified and the mean value of each group was presented in the bar figure under the image. (**C**) mRNA of MECR in liver. mRNA was determined by qRT-PCR. Each bar represents mean ± SD (*n*=6). **,*P*<0.01; ***, *P*<0.001.

The mechanism of tissue-specific regulation was unknown for MECR protein. To address this question, we examined MECR protein in the liver of DIO mice in exploration of the mechanism. Liver suffers insulin resistance in the DIO mice as indicated by the value of insulin resistance index HOMA-IR (Figure 3A). There is no report about the relationship of MECR and the hepatic insulin resistance in the DIO mice. The MECR protein was examined in the tissue homogenization of liver by Western blotting. A significant decrease was observed in the DIO mice (Figure 3B). The reduction was associated with an increase in mRNA of MECR (Figure 3C), which suggests that the turnover rate of MECR protein is increased in the liver of DIO mice for the fall in protein abundance. The protein fall is associated with insulin resistance.

### Restoration of MECR protein in DIO mice by BBR

**Figure 4:**
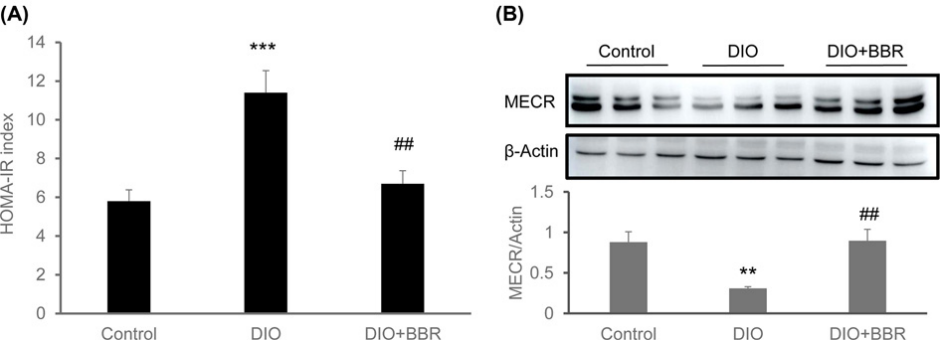
Restoration of MECR protein in liver of DIO mice by BBR (**A**) Insulin sensitivity. The insulin resistance index of HOMA-IR was calculated with fasting insulin and fasting blood glucose of DIO mice after 8-week-treatment with BBR (100 mg/kg/day) (*n*=9). (**B**) BBR effect. The MECR protein was examined in the liver tissues of DIO mice after BBR treatment for 8 weeks. The data are presented as mean ± SD (*n*=6). **, *P*<0.01; ***, *P*<0.001 vs. control; ^##^, *P*<0.01 vs. DIO.

It is interesting to observe the association of MECR protein reduction and insulin resistance above. The observation suggests that MECR protein recover when the insulin resistance is corrected. To test the possibility, we examined MECR protein in the liver of DIO mice treated with BBR. BBR is a compound with an activity in the improvement of insulin sensitivity [21]. The insulin resistance was improved in the BBR-treated DIO mice (Figure 4A). The MECR protein was restored in the liver of BBR-treated mice (Figure 4B). The data suggest that MECR protein recovers in the liver by restoration of insulin sensitivity in DIO mice. The data provide evidence for BBR regulation of MECR protein in the liver of DIO mice.

### Reduction in MECR protein in response to ATP

**Figure 5:**
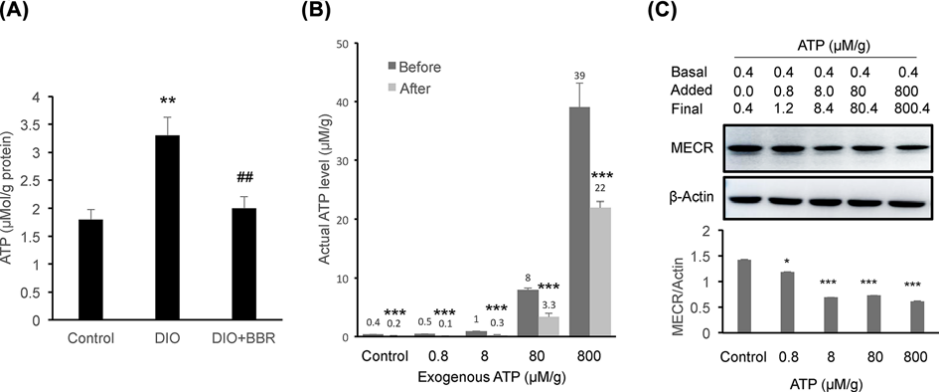
Inhibition of MECR protein by ATP (**A**) ATP level in the liver of DIO mice. The ATP level was examined in fresh liver tissue of DIO mice. (**B**) ATP concentrations in the cell lysate before and after incubation. The cell lysate of 1c1c7 cells was used after overnight storage at −80°C to reduce the endogenous ATP. The catual ATP (μM/g) concentration was measured in the lysate with normalization to protein after the ATP addition. The actual concentration number is shown at the top of each bar. Comparison was made before and after the incubation to determine ATP reduction by hydrolysis. (**C**) MECR protein reduction in response to exogenous ATP. The MECR protein was determined after 15-min incubation at 37°C with ATP at different concentrations that are indicated at the top of each lane. The protein was determined by Western blotting with quantified signals in the bar figure. The ATP levels calculated in different ways are shown on the top of each lane in the blot. Exogenous ATP level is shown in x-axis of the bar figure. The data in bar figure represent mean ± SD (*n*=3). *, *P*<0.05; **, *P*<0.01; ***, *P*<0.001 over the control; ^##^, *P*<0.01 over the DIO by Student’s *t* test.

To investigate the mechanism of MECR reduction in DIO mice and recovery by BBR, we focused on ATP. ATP is produced primarily by mitochondria and glycolysis in the cytosol. The production is driven by substrate supply including glucose, fatty acid and oxygen, etc. Intracellular ATP is elevated in the liver of DIO mice due to the increase in glucose/fatty acid supply [2]. The intracellular ATP is reduced by BBR through suppression of mitochondrial function [20]. The data suggest that the MECR protein reduction may be a result of ATP elevation in DIO mice, and its recovery is a result of disappearance of the ATP elevation by BBR treatment. To test the possibility, ATP level was examined in the liver of BBR-treated DIO mice. The elevation was observed in DIO mice, which was blocked by BBR treatment of the DIO mice (Figure 5A). The observation suggests a role of ATP in the regulation of MECR protein.

To test the ATP activity further, MECR protein was examined in the whole cell lysate with an increase in the ATP concentration. The test was conducted by addition of exogenous ATP to reach higher concentrations in the lysate. The basal ATP level was reduced to 0.4 μM/g in the cell lysate by overnight storage at −80°C, which was done to decrease the ATP background to allow increase in the ATP level through addition of exogenous ATP. When ATP was added into the cell lysate, the ATP level was decreased quickly as indicated by the data in Figure 5B. The theoretical concentrations are indicated in x-axis and the actual concentrations are indicated in y-axis. The lysate was incubated in 37°C water bath for 15 min to allow ATP to generate an impact in the protein. ATP was determined in the lysate before and after the incubation following ATP addition to understand the ATP activity. The actual ATP level was much lower than the theoretical level (Figure 5B), suggesting a quick hydrolysis of ATP in the lysate. After the incubation, the ATP level was further decreased with more than 50% loss. These data support that ATP is unstable in the cell lysate and subject to quick hydrolysis. The ATP hydrolysis was associated with MECR protein degradation (Figure 5C). The MECR protein was significantly reduced in the cell lysate when the exogenous ATP was added to the theoretical level of 1.2 μM/g. More reduction was observed at the theoretical level of 8.4 μM/g. There was no further MECR reduction at concentrations above 8.4 μM/g. These data suggest that the MECR protein is very sensitive to the elevation of ATP with a response of protein degradation at ATP concentrations between 0.4 and 8.4 μM/g.

## Discussion

The present study provides a new insight into the mechanism by which ATP induces insulin resistance. The insight is identification of the LA synthetic pathway in the feedback regulation of mitochondria by ATP. In the present study, ATP was found to inhibit MECR protein in the LA synthetic pathway in three models: DIO mice, cellular model and BBR model. The increase in intracellular ATP is associated with insulin resistance in several tissues of DIO mice [2,18,19] and in liver of healthy individuals by overfeeding [22]. The role of ATP in the pathogenesis of insulin resistance is supported by the activities of metformin [3] and chemical uncouplers [23,24], both of which improve insulin sensitivity *in vivo*. Metformin reduces ATP production through suppression of the respiratory chain in mitochondria. The chemical uncouplers reduces ATP production through reduction in the mitochondrial potential. However, the molecular mechanism of ATP action is elusive in the insulin resistance. Current study suggests that MECR may mediate the ATP signal in the DIO mice for insulin resistance. MECR is required for the maintenance of mitochondrial function [13,14]. MECR reduction may lead to mitochondrial malfunction in the glucose metabolism, which represents a new mechanism of insulin resistance. The MECR protein reduction by ATP elevation suggests a novel mechanism for ATP feedback regulation of mitochondria.

The mechanism of MECR protein reduction remains unknown in response to ATP. It may involve in the post-translational modification of MECR protein by phosphorylation or other types of modifications. Mitochondrial proteins are subject to post-translational modification including phosphorylation, acetylation, succinylation etc. [25]. The modifications are catalyzed by enzymes, whose activities are dependent on ATP in energy supply. Their activities are induced by elevation of the substrate ATP. However, it is unknown if those enzymes contribute to the MECR response to ATP. The possibility remains to be tested.

Current study demonstrated that MECR mRNA was induced by insulin and decreased by the cAMP/PKA activator (forskolin) in the mammalian cells. MECR function has been investigated in the yeast and mammalian cells. However, there is no report about the MECR regulation in the physiological conditions. One study reported that *Mecr* expression was regulated by miR-125b in adipocytes [26], but the physiological significance remains unknown. Our observation suggests that *Mecr* expression may be regulated by insulin and glucogan (cAMP/PKA) in the physiological conditions. The observation provides a mechanism for *Mecr* alteration in the obese mice under hyperinsulinemia. The *Mecr* mRNA was elevated in the liver of DIO mice in the present study, which was likely a consequence of hyperinsulinemia in the DIO mice according to the insulin activity in the induction of *Mecr* mRNA in the cell culture model.

MECR may represent a new target for BBR activity in the improvement of insulin sensitivity. It is generally believed that BBR enhances insulin sensitivity through down-regulation of ATP, which in turn leads to activation of AMPK signaling pathway [21]. AMPK enhances mitochondrial function through multiple pathways, such as mitochondrial biogenesis and mitophagy etc. [27]. Except AMPK, there is little information about mechanism of BBR impact in mitochondria. BBR restored the MECR protein level in the liver of DIO mice in current study, which provides a new mechanism for the BBR activity in the regulation of insulin sensitivity. However, this possibility remains to be tested with a *Mecr* knockout model.

In summary, the present study identified physiological signals (insulin, cAMP/PKA and ATP) and one medicine (BBR) in the regulation of MECR expression. The observations provide a mechanism for ATP in the feedback regulation of mitochondria. Inhibition of MECR protein by ATP suggests a novel mechanism for feedback regulation of mitochondrial function with potential implication into the mechanism of insulin resistance. MECR may serve as a new link for regulation of mitochondrial function by insulin and the cAMP/PKA pathway.

## Abbreviations

AMPK: AMP-activated protein kinase
BBR: berberine
DIO: diet-induced obese
FAS II: fatty acid synthase II
HOMA-IR: homostatic model assessment of insulin resistance
LA: α-lipoic acid
MCAT: malonyl-CoA-acyl carrier protein transacylase
MECR: mitochondrial 2-enoyl-acyl-carrier protein reductase
mtFAS: mitochondrial fatty acid synthase
OXSM: 3-oxoacyl-ACP synthase of mitochondria
PKA: protein kinase A
qRT-PCR: quantitative reverse transcription-PCR
SPF: specific-pathogen-free
